# Public School Athletics

**Published:** 1914-05

**Authors:** 


Public School Athletics.—Many of the high schools of the Southwest are now regularly employing trained physical directors and supervisors of the playgrounds. This step marks a new era in school work, for it gives distinct direction to the physical development of high school students. The average high school boy or girl is already physically alert, a bunch of energetic muscles and nerves, that should be properly trained and developed. It is known that proper physical training is as essential to success as correct mental training. Heretofore physical development has largely been left to chance, or at least it has been without any studied direction. The physical directors will be men who understand anatomy and physiology as well as physical education, who have taken courses in the physiology of bodily exercises, drill regulations, educational play, corrective gymnastics, physical and medical examinations, first aid to the injured, etc. Besides teaching how to play they must teach the children those plays that will best develop their bodies and cure any physical defects, the plays that will make strong men and women of them. Many boys are playing football who are apparently well and strong, but whose physical conditions are such that they should engage only in less vigorous plays, and it is known that basket ball and some other sports for girls are too great a tax upon the constitution of many girls. Physical direction will be welcomed by all parents who are interested in the proper development of their children.

